# Quantification of Thread Engagement in Screw-Plate Interface of Polyaxial Locking System Using X-ray Computed Tomography

**DOI:** 10.3390/ma16175926

**Published:** 2023-08-30

**Authors:** Tomasz Bartkowiak, Daria Madalińska, Patryk Mietliński, Jakub Kaczmarek, Bartosz Gapiński, Marcin Pelic, Piotr Paczos

**Affiliations:** 1 Institute of Mechanical Technology, Poznan University of Technology, 60-965 Poznań, Poland; daria.mad2021@gmail.com (D.M.); patryk.mietlinski@put.poznan.pl (P.M.); bartosz.gapinski@put.poznan.pl (B.G.); marcin.pelic@put.poznan.pl (M.P.); 2 VETACARE—Dr. Philipp Winkels, 50374 Erftstadt, Germany; vet.kaczmarek@gmail.com; 3 Institute of Applied Mechanics, Poznan University of Technology, 60-965 Poznań, Poland; piotr.paczos@put.poznan.pl

**Keywords:** thread engagement, orthopedics, X-ray computed tomography, polyaxial locking systems

## Abstract

This study demonstrates a new method for quantifying thread engagement in mechanical connections and verifies its applicability using biomedical implants under push-out tests. The focus is on orthopedic plate implants employed for bone fracture fixation, which, by design, allow off-axis screw insertion to accommodate different contact conditions. Thread engagement is crucial in determining connection strength and stability. In medical practice, off-axis screw placement is usually necessary due to bone geometries and implant plate rigidity. To address this, the study proposes a quantification method using non-destructive testing with X-ray micro-computed tomography and automated image processing, although tuning the image processing parameters is vital for accurate and reliable results. This enables detailed 3D models of screw-plate interfaces for precise thread engagement measurement. The results show that thread engagement decreases with both, increased off-axis insertion angles and higher torque during insertion. Correlation analysis reveals a strong relationship (R^2^ > 0.6) between average thread engagement and push-out strength, underscoring the importance of proper screw placement for stable fixation.

## 1. Introduction

The purpose of this study is to explore the capabilities of the new method that enables the quantification of thread engagement in mechanical connections and to determine correlations between the actual screw-plate thread engement and strength of that interface in biomedical implants under destructive single-cycle-to-failure push-out test. The method has been primarily developed for orthopedic plate implants which are used for bone fracture fixation and allow off-axis screw insertion resulting in achieving different contact conditions. 

The development of angle-stable locking screw fixation was one of the most significant advances in bone plate osteosynthesis of fractures [1,2,3]. The stability of the angle-stable plates depends on accurate screw insertion, their design, and the strength of the interlock between screw head and the plate hole. Advantages of locking plate fixation include the reduced need for accurate anatomical plate contouring, improved construct stability in osteoporotic bone and maintenance of bone blood supply. However, the requirement to insert locking screws at a predetermined fixed angle to the bone plate can be very difficult or even impossible at some locations [4]. This problem can occur at the point when intraoperatively a straight locking plate will be bent to fit the bone anatomy resulting in directing the locking screw into the articular surface. A recent development to overcome these limitations was the introduction of polyaxial locking systems, which allow screw insertion at different angles while still offering the advantages of a locked construct [5,6]. Insertion of the screw abaxially changes the interface between threaded components what can potentially affect the performance of the implant [7]. 

In engineering, thread engagement is a crucial metric while selecting fasteners, as it directly affects the strength and stability of the connection [8,9]. While making a connection, the bolted joints should be designed so that the screw breaks before the threads strip. For components with tapped holes, the length of engagement (the number of threads engaged between the tapped hole and the screw) should be adjusted to favor this criterion. The thread engagement is determined depending on the thread type, pitch, nominal diameter and material of both connecting parts. Thread engagement plays the fundamental role in the performance of the connection and the mode of failure [10]. In engineering, the abaxial placement of the screw is rare and often due to the bad design or manufacturing, while in human and veterinary medicine it is common clinical practice due to the complex geometry of bones and the rigidness of implant plates.

The quantification of thread engagement in screw-plate interface can be performed using non-destructive testing that involves X-ray micro-computed tomography. Kaczmarek et al. developed a method which used automated image processing of 2D scans which were derived from 3D scanned model as captured by metrological micro-CT scanner. They applied that technique to determine differences in average thread engagement in polyaxial and monoaxial veterinary locking plates which were conducted to a static and cyclic loading [11]. According to authors’ best knowledge there are no other existing experimental methods that allow measurement of thread engagement in screw-plate interface.

X-ray micro-computed tomography is a technique that is widely used in the investigation of bone-implant-related biomechanical problems [12,13]. The majority of the published research focus on the interface between screw and bone [14]. The motivation behind this is the recognition that maintaining sufficient compression between the threads of a screw and the neighboring bone is crucial for ensuring stable fixation [15]. Since this primarily depends on cancellous bone and has limited cortical support in the pedicle area [16], stability can be compromised due to the low density of the bone surrounding the implant [17]. Chevalier et al. created micro finite element (µFE) models to evaluate the specific impact of screw design and the relative role of local bone density in fixation mechanics [15]. These models were generated from micro computer tomography (µCT) scans of vertebras that had been implanted with two different types of pedicle screws. The authors emphasized the significance of local density within the pedicle region in relation to the stiffness and strength of pedicle screws during pull-out loads. Similar approach was presented in [14]. Wirth et al. applied microCT and numerical analysis to verify if peri-implant bone microstructure affects implant stability in trabecular bone, to a greater degree than more distant bone [18]. Torcasio et al. quantified peri-implant bone strains after the implant placement in rat tibiae [19]. In other work, Steiner et al. focused on the prediction of primary implant stability of screws in bone considering also insertion-related bone damage [20].

Apart from applying 2D or 3D bone models in the numerical simulations, microCT has been also used in analyzing the osseointegration as the untightening of the prosthetic screw is the most common problem in oral rehabilitation with implants. Batista et al. determined that the torque applied is crucial to the head deformation in titanium screws which fix the prostheses to the implant [21]. In other study, authors used microCT analysis to determine the effect of fixing grafted bone with an adhesive, screw and their combination. The observed no differences in the osseointegration of implants placed in grafted autogenous bone blocks fixed with cyanoacrylate-based adhesive and screws [22]. The presence of resin embedment, and implant configuration can have the impact on the occurrence of the measurement artifacts and the quality of resulted 3D geometry. Li et al. provide that implant angulation and resin embedment affected the artifact volume of micro-CT scanning for dental implant [23].

The presented research addresses the problem of quantifying the thread engagement in screw-plate interface using micro-CT measurements and automated image analysis. The method was applied to study the effect of off-axis insertion on the average thread engagement. This measure was correlated with the push-out strength of the screw from the plate. This paper should contribute to a better understanding of the locking process in orthopaedic implants. 

## 2. Materials and Methods

### 2.1. Screw-Plate Constructs

The subject of our study is polyaxial locking system plate (PLS) (Aesculap B. Braun Vet Care, Tuttlingen, Germany). Both plate and screw were made of 316 stainless steel. The locking screws have a 3.5-mm thread and a 2.6-mm core diameter. This locking plate system allows abaxial locking screw insertion in various non-perpendicular directions. The manufacturer recommends a maximum deviation angle away from the perpendicular of 15 degrees. To achieve locked fixation, the screw head engages into five threaded contact regions inside the plate hole depending on the insertion angle.

**Figure 1:**
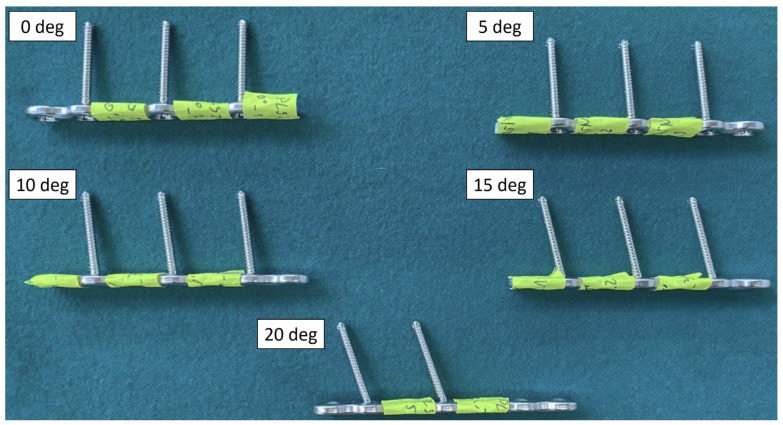
Overview of the construct angular configurations: screws inserted at 0, 5, 10, 15 and 20 degrees off-axis. Please note that the locking screws had a 2.6 mm core and 3.5 mm thread diameter and length of 36 mm. They were placed in a seven-hole PLS plate in hole no. 2, 4 and 6, with one plate hole left empty between the screws. The plate length was 84 mm.

To determine the effect of different off-axis insertion of the screw, which hypothetically can lead to differences in thread engagement, the screws were locked at 0, 5, 10, 15 and 20 degrees in off-axis configurations (Figure 1). The additional factor of tightening torque of 1.5 and 2.5 Nm was added which could potentially affect the screw-plate threaded interface. The size of each group was equal to *n* = 4. In this paper we used the same dataset that was originally published in authors’ previous study [7], which focused on the mechanical performance only, without considering the aspect of thread engagement.

### 2.2. Quantification of Thread-Engagement with Microct and Automated Image Processing

To quantify the amount of thread engagement we use the new possibilities offered by X-ray micro computed tomography which is a 3D imaging technique utilizing X-rays to measure the geometry of parts, slice by slice with high resolution. In this paper, metrological computed tomographic (v|tome|x s240, GE Sensing & Inspection Technologies GmbH, Wunstorf, Germany) data from measurement of screw head—plate hole interfaces 3.5 mm system PLS as described by Kaczmarek et al. [11]. The scanning parameters used for the metrological CT scans were: beam power of 25.3 W (230 kV/110 μA), exposition time for a single picture 350 ms and the voxel size 10.6 μm.

**Figure 2:**
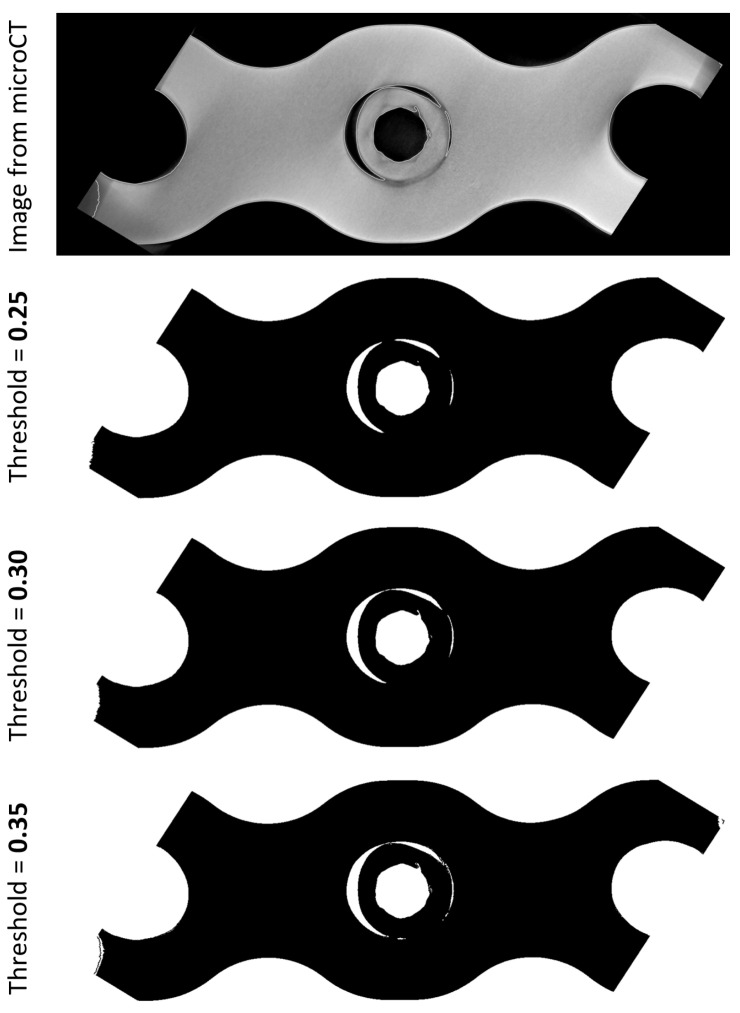
The effect of threshold on the binarized representation of microCT images of the thread interface.

The procedure to calculate average thread engagement (ATE) is divided into 5 steps [11]. In short, following micro-CT measurement, a 3D model of each threaded connection was created, and it was later transformed into a series of photos representing cross-sections that were taken every 50 μm orthogonally to the locking screw axis. In those 2D images, thread engagement was evident as a region devoid of a recognizable space between the screw and the plate. The contact or gap was found using the automated image processing, which involved, apart from other methods, image binarization using Canny edge detection. The method transforms color images into black and white images, where black represents material and white is the gap. This binarization takes into account the threshold parameter (from 0 to 1) which should be adjusted for the individual application. In this work, we investigated three arbitrarily selected different threshold values 0.25, 0.30 and 0.35. Threshold impacts the presence or absence of the regions for which contact between screw and plate thread can be detected (Figure 2).

**Figure 3:**
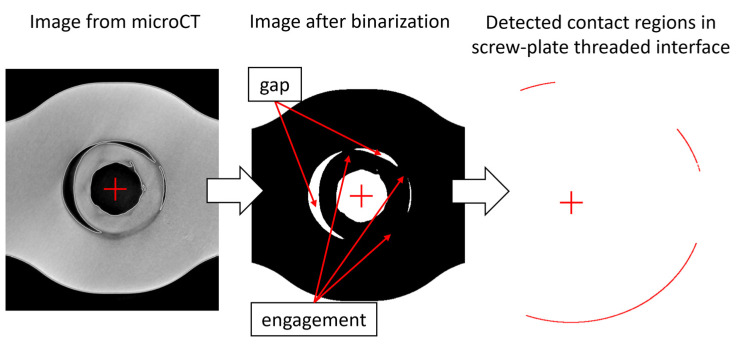
From grayscale image from microCT representing thread engagement between screw and plate, through binarized image in which evident gap and engagement regions (marked with red arrows) can be seen, to the results of the algorithm indicating three regions for which contact between screw and plate is detected.

For each analyzed image, it was assumed that there was a full engagement where there was no space between the screw and the plate. Each image received a score between 0% and 100% based on how much contact was determined by automatically examining the screw-plate interface region pixel by pixel. The average thread engagement (ATE) measure, which was calculated for all cross-sections, represents the arithmetic mean of the individual scores obtained for all analyzed images. The thread-to-thread contact regions of each screw head was visualized in a form of three-dimensional polar plot. Figure 3 shows the principles of the algorithm from grayscale image from microCT to the detected regions for which contact between screw and plate is evident. The entire algorithm was implemented in Mathematica 12 (Wolfram Research, Inc., Champaign, IL, USA).

### 2.3. Relationship between ATE and Push-Out Strength

The proposed method is used here to determine the relationship between ATE and the performance of the screw-plate interface using push-out strength. To evaluate the push-out strength of the locked screws in the two locking systems, each plate was firmly secured into a custom-made jig, and only the hole with the screw inserted was left accessible. The tip of the screw pointing toward the actuator was engaged by a concave surface. The push-out force was applied in the axial direction at a loading speed of 0.1 mm/s and was recorded at 10 Hz. Destructive static tests were performed using a servo-hydraulic testing machine (ZWICK Z100/TL3S, Zwick GmbH & Co. KG, Ulm, Germany) with a measuring range of up to 100 kN and fitted with a mechanical drive. The tests were performed under displacement control. 

### 2.4. Statistical Analysis

Data are reported as mean ± standard deviation. Shapiro–Wilk tests were performed to test for the normal distribution of residuals. For the studied insertion torques and angles two-way ANOVA was performed to determine whether these factors affected the average thread engagement. Post hoc Bonferroni tests were conducted to determine whether there were any statistically significant differences between the groups. Regression analysis was performed independently for each insertion torque. The coefficient of determination was calculated to indicate the strength of the correlation between the ATE and push-out strength. Statistical analyses were performed using Mathematica 12 software (Wolfram Research, Inc., Oxfordshire, UK). Statistical significance was set at a *p*-value of <0.05.

## 3. Results

### 3.1. MicroCT Scans

**Figure 4:**
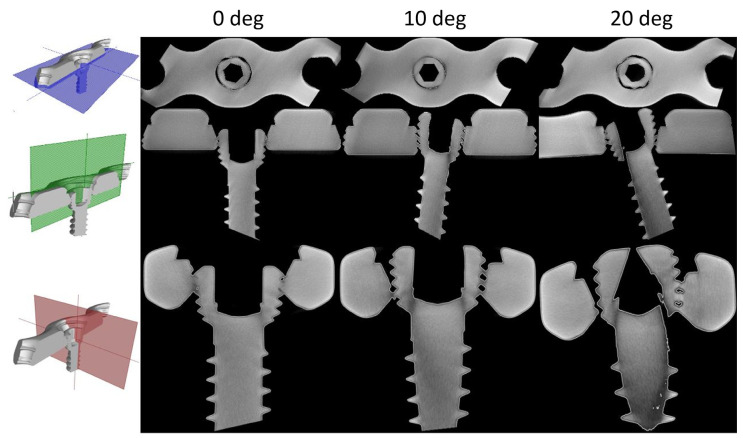
Overview of the microCT scanned screws inserted into the plate at 0, 10 and 20 degrees off-axis. Please note that each row corresponds to the images depicting a cross-section views referring to the different cutting planes (first column).

Figure 4 presents 2D images representing cross-sections created from the acquired 3D models of screws inserted abaxially into the plate at 0, 10 and 20 degrees. The interface between threaded components can be seen as region of similar color, while black represents void or no contact. The thread engagement differs between varying insertion angles. Further investigation was conducted for images taken perpendicularly to the scr axis, as presented in Figure 4 (top row).

### 3.2. Thread Engagement

Statistical analysis revealed that locking torque and angle are relevant parameters affecting ATE (*p* < 0.001). Image processing parameter—threshold is also influencing the ATE (*p* < 0.001). The combinations between torque, angle and threshold were not found to be significant factors (*p* > 0.005). The results are presented in Table 1 as mean ± standard deviation calculated for each of the analyzed groups.

**Figure 5:**
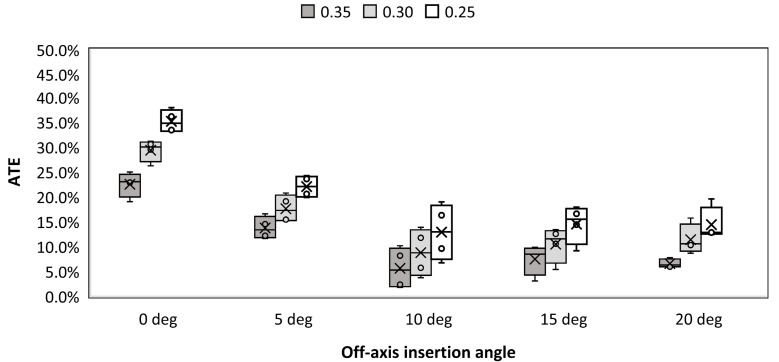
Average thread engagement (ATE) vs. off-axis insertion angle for three different threshold values and torque equal to 1.5 Nm.

**Figure 6:**
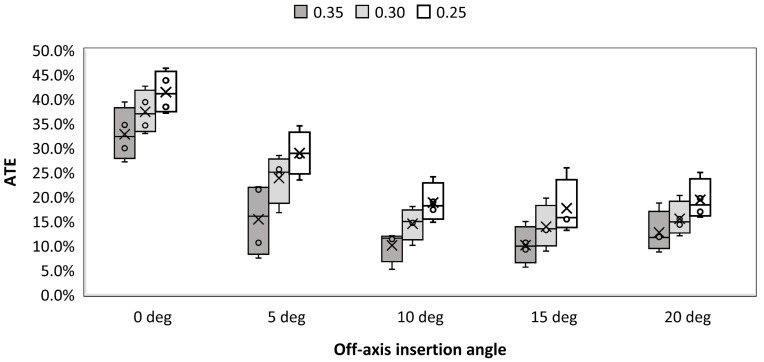
Average thread engagement (ATE) vs. off-axis insertion angle for three different threshold values and torque equal to 2.5 Nm.

**Figure 7:**
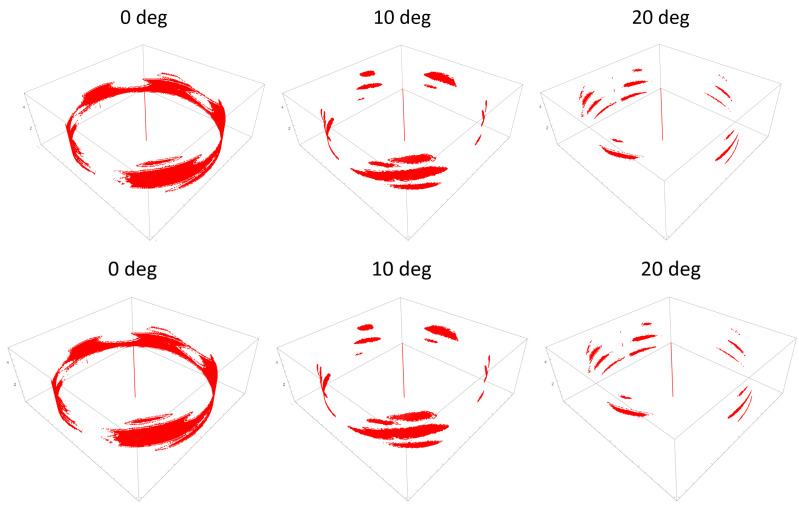
Graphical representation of thread engagement for three representative interfaces for which screw was inserted at 0, 10 and 20 degree and using 1.5 Nm tightening torque.

Figure 5 presents the evolution of ATE as a function of off-axis insertion angle for three different thresholds and locking torque equal to 1.5 Nm. It can be seen that ATE decreases with an insertion angle between 0 and 10 degrees. Further increase of ATE does not contribute to the significant change of ATE. Lowering the threshold in image binarization leads to the increase of estimated ATE. This is caused by the fact that the more portions of the gray area on the images are considered actual material what increases detected engagement. A similar observation can be made for samples which were locked using 2.5 Nm torque (Figure 6). Higher value of torque also contributed to the increase of ATE when compared to 1.5 Nm. Visual representation of regions where contact was detected for screws inserted at 0, 10 and 20 degrees off-axis and locked with 2.5 Nm is depicted in Figure 7. An evident change in thread engagement can be noted with increasing off-axis insertion angle.

### 3.3. Correlations with Push-Out Strength

**Figure 8:**
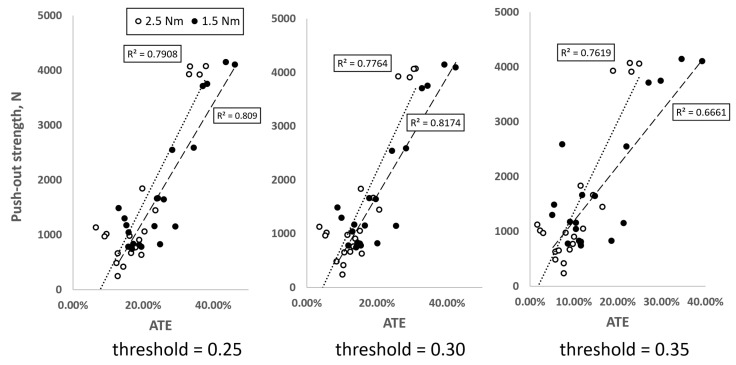
Push-out strength vs. ATE for two different torques and three different threshold values.

The relations between push-out strength and ATE were presented in Figure 8 for three different threshold values and two considered torques. When regressed linearly, strong correlations (R^2^ > 0.6) between those two quantities can be seen for all analyzed threshold values. The highest coefficient of determination can be seen for threshold equal to 0.3 and 1.5 Nm torque (R^2^ > 0.817). The highest threshold produced least correlated results for the lowest torque. For torque equal to 2.5 Nm the strongest correlations can be observed at the lowest threshold (R^2^ > 0.809). The medium considered value of threshold equal to 0.3 appeared to be most convenient in terms of finding the strongest relations. 

## 4. Discussion

Forces acting on the fracture after its fixation during ambulation are a combination of bending, axial compression, torsion, and shear forces. From a clinical point of view, the push-out forces achieved in this experiment are likely to be sufficient to maintain the screw-plate connection until the bone has healed. However, the exact values of the force acting on the locking plate system in vivo have not been determined yet. Additionally, is still not known how much force is delivered to a single screw during ambulation and how much connection is needed to retain a stable fixation between the screw head and the plate hole. Another point worth mentioning is that a minimal number of locking screws per fracture segment should be two screws which fully penetrates the bone on both ends. As a result, the stress and strain will be delivered over the screws and the plate accordingly to its location and the working length. 

Placing the locking screw in an off-axis fashion is a very tempting option especially when a juxta-articular fracture is being treated. This is because the intraoperative bending of locking plates follows the curved contour of the metaphysis which can inadvertently direct fixed-angle locking screws into the articular surface. There are several solutions to this problem:

- a shorter screw can be used,
- a plate hole can be left empty,
- a cortical screw can be used,
- a polyaxial locking plate can be used instead of a fixed-angle locking plate,
- intentionally use a fixed angle locking screw in an off-axis position.

Each of those options might reduce the strength of the plate construct in this region. Our study demonstrated that a small deviation in a screw insertion angle in a fixed-angle locking system leads to a significant drop in its holding strength and ATE and therefore should be avoided. 

Another point worth mentioning is an increase of push-out strength when the insertion torque has been set on a higher value. Although higher ATE was obtained when locking at 2.5 Nm, this practice also cannot always be recommended because it may theoretically lead to cold welding and difficulties in later extraction of the screw from the plate [7].

From the engineering point of view, it was shown that ATE was affected by the insertion angle and it decreased together with off-axis angle regardless of the automated image processing threshold value selected. The actual percentage of thread engagement is difficult to measure directly as both threaded parts are locked in each other. The application of metrological microCT allowed the measurement of the thread interface of screw and plate when engaged. Following the automated image processing of the images derived from 3D representation, it was possible to determine the ATE directly in the locked state.

In the medical literature related to the threaded components the main focus in given into the interface between screw and bone [15,19,24,25,26]. This is due to the fact that the strength and the stability of the construct is affected by the material and microstructure of the bone the screw cuts into. This paper addresses the second factor which is the interface between screw and plate, which is important especially for screw inserted abaxially. Following the path of the development of µFE models of bone-screw interfaces, a numerical model for screw-plate threaded connection can be potentially developed. This would allow connecting any practical case with the bone stress state. This could also facilitate the analysis of other loading conditions: shearing, bending or torsion.

The lack of other method which can experimentally estimate ATE in threaded connections does not provide any benchmark we can refer to when validating our results. However, a strong correlation found between push-out strength and ATE proves the pertinence of the proposed method. Although the results were affected by the threshold value, still similar relations between the two aforementioned quantities can be found. Note that the three analyzed threshold values were selected arbitrarily from range between 0 (almost entirely dark image after binarization) and 1 (almost entirely white image after binarization). This was conducted by visual inspection of the images being produced by the algorithm and for threshold lower than 0.25 or greater than 0.35 it was either a situation in which contact was detected in regions where it did not appear to exist or some information about the apparent engagement was lost. This research does not provide an answer to the question what the most convenient threshold is because it will depend on the particular case and the grayscale representation of the microCT scans. It is authors’ intention to draw the attention that tuning of the image processing parameters is an important aspect of any study involving similar algorithms. Proper selection of the parameters is always important in order to achieve high quality of the results. 

## 5. Conclusions

Overall, this research contributes to a better understanding of the mechanics involved in screw-plate interfaces for orthopedic implants. The proposed method enables the quantification of thread engagement, providing valuable insights into the locking process and implant performance. The main conclusions of this study focused on PLS can be summarized in the following points:

- ATE is affected by the insertion angle and locking torque. Thread engagement decreases with off-axis insertion angle and increases with applied torque,
- push-out strength strongly correlates with ATE but the strengths of correlations are affected by the threshold, so careful adjustment of the image processing parameters is important.

The findings can inform surgical practices, enhancing the design and placement of orthopedic implants for improved patient outcomes. By shedding light on the mechanical behavior of threaded connections, this study addresses a critical aspect of biomedical engineering and offers valuable guidance for future implant developments.

A potential continuation of this research can involve development of the FEM-based model of the interface considering actual geometry of screw and plate threaded and off-axis insertion. That model, if capable of providing the right push-out correlation to engagement, would allow to connect numerically any practical case with the bone stress state. This can also help to analyze other loading conditions: shearing, bending or torsion. 

## Figures and Tables

**Table 1:** ATE calculated for different threshold values. The results are presented as mean ± standard deviation.

Torque	Insertion Angle	ATE		
		Threshold = 0.25	Threshold = 0.30	Threshold = 0.35
1.5 Nm	0 deg	35.16% ± 2.28%	29.37% ± 2.2%	22.51% ± 2.51%
	5 deg	22.06% ± 2.17%	17.61% ± 2.77%	13.72% ± 2.27%
	10 deg	12.89% ± 5.7%	8.77% ± 4.82%	5.62% ± 4.18%
	15 deg	14.52% ± 3.86%	10.47% ± 3.59%	7.46% ± 3.04%
	20 deg	14.41% ± 3.43%	11.37% ± 3.05%	6.56% ± 0.87%
2.5 Nm	0 deg	41.34% ± 4.37%	37.33% ± 4.43%	32.73% ± 5.39%
	5 deg	28.88% ± 4.53%	23.77% ± 5.01%	15.36% ± 7.47%
	10 deg	18.77% ± 3.92%	14.45% ± 3.29%	10.02% ± 3.26%
	15 deg	17.6% ± 5.67%	13.83% ± 4.46%	10.06% ± 3.86%
	20 deg	19.31% ± 4.08%	15.46% ± 3.48%	12.65% ± 4.26%

## Data Availability

The research data is available upon request.
